# Computer-aided diagnosis of chest X-ray for COVID-19 diagnosis in external validation study by radiologists with and without deep learning system

**DOI:** 10.1038/s41598-023-44818-9

**Published:** 2023-10-16

**Authors:** Aki Miyazaki, Kengo Ikejima, Mizuho Nishio, Minoru Yabuta, Hidetoshi Matsuo, Koji Onoue, Takaaki Matsunaga, Eiko Nishioka, Atsushi Kono, Daisuke Yamada, Ken Oba, Reiichi Ishikura, Takamichi Murakami

**Affiliations:** 1 Department of Radiology, Kobe University Graduate School of Medicine, 7-5-2 Kusunoki-Cho, Chuo-Ku, Kobe, 650-0017 Japan; 2 Department of Radiology, St. Luke’s International Hospital, 9-1 Akashi-Cho, Chuo-Ku, Tokyo, 104-8560 Japan; 3 Department of Radiology, Kobe City Medical Center General Hospital, 2-1-1 Minatojimaminamimachi, Chuo-Ku, Kobe, 650-0047 Japan; 4 Department of Diagnostic Imaging and Interventional Radiology, Kyoto Katsura Hospital, 17 Yamada-Hirao, Nishikyo-Ku, Kyoto, 615-8256 Japan

**Keywords:** Diseases, Infectious diseases, Bacterial infection, Viral infection, Mathematics and computing, Software

## Abstract

To evaluate the diagnostic performance of our deep learning (DL) model of COVID-19 and investigate whether the diagnostic performance of radiologists was improved by referring to our model. Our datasets contained chest X-rays (CXRs) for the following three categories: normal (NORMAL), non-COVID-19 pneumonia (PNEUMONIA), and COVID-19 pneumonia (COVID). We used two public datasets and private dataset collected from eight hospitals for the development and external validation of our DL model (26,393 CXRs). Eight radiologists performed two reading sessions: one session was performed with reference to CXRs only, and the other was performed with reference to both CXRs and the results of the DL model. The evaluation metrics for the reading session were accuracy, sensitivity, specificity, and area under the curve (AUC). The accuracy of our DL model was 0.733, and that of the eight radiologists without DL was 0.696 ± 0.031. There was a significant difference in AUC between the radiologists with and without DL for COVID versus NORMAL or PNEUMONIA (*p* = 0.0038). Our DL model alone showed better diagnostic performance than that of most radiologists. In addition, our model significantly improved the diagnostic performance of radiologists for COVID versus NORMAL or PNEUMONIA.

## Introduction

The novel coronavirus disease 2019 (COVID-19), a new infectious disease, was first discovered in China in 2019 and has currently caused a significant number of infections and deaths worldwide^1^. At the time of writing this paper, a total of at least 529,410,287 infections and 6,296,771 deaths have been confirmed worldwide^2^. The development of vaccines and measures to prevent the spread of the disease have temporarily succeeded in reducing the number of infected people. However, the threat of COVID-19 continues worldwide because of a highly infectious species known as the Omicron strain.

Real-time polymerase chain reaction (RT-PCR) is used as a diagnostic method for COVID-19 in many medical institutions. However, RT-PCR is not always an effective method. One report has indicated that computed tomography (CT) is more sensitive than RT-PCR^3^. CT and chest X-ray (CXR) may serve as more accurate diagnostic methods for COVID-19^4,5^.

The clinical application of deep learning (DL) in the diagnosis of COVID-19 on CXR has attracted attention^6,7^. Although CXR is less accurate than CT, CT scanners are not always available. For example, as a 24/7 in-hospital service, rural hospitals have very limited local access to CT scanners^8^. CXR is simple and inexpensive, and radiation exposure of CXR is less than that of CT. Therefore, if COVID-19 can be diagnosed using a combination of DL and CXR, it may be possible to screen for COVID-19.

Many studies have already been conducted on CT/CXR for the diagnosis of COVID-19 using DL, and most of them have shown promising results^9–11^. However, in the case of the clinical application of DL as a computer-aided diagnosis system, medical doctors must compare their own diagnosis with that of DL. If there is an inconsistency between doctors and DL, doctors may reject the DL diagnosis. To evaluate the clinical usefulness of DL, an observer study of CXR readings must be conducted for both DL and radiologists. Only a few studies have compared the diagnostic performance of DL and radiologists^12–14^.

This study aimed to evaluate the diagnostic performance of our DL model of COVID-19 and investigate whether radiologists changed their diagnosis by referring to our DL model of CXR and whether the diagnostic performance of radiologists was significantly improved. To evaluate the clinical usefulness of DL, an observer study of radiologists and external validation of our DL model were conducted. Based on the reading sessions of the observer study, the diagnostic performance was compared among (i) our DL model, (ii) eight radiologists without DL, and (iii) eight radiologists with DL.

## Materials and methods

This retrospective study was approved by the institutional review boards of eight hospitals (Kobe University Hospital, St. Luke's International Hospital, Nishinomiya Watanabe Hospital, Kobe City Medical Center General Hospital, Kobe City Nishi-Kobe Medical Center, Hyogo Prefectural Kakogawa Medical Center, Kita Harima Medical Center, and Hyogo Prefectural Awaji Medical Center); the requirement for acquiring informed consent was waived by the institutional review boards of these eight hospitals owing to the retrospective nature of the study. This study complied with the Declaration of Helsinki and Ethical Guidelines for Medical and Health Research Involving Human Subjects in Japan (https://www.mhlw.go.jp/file/06-Seisakujouhou-10600000-Daijinkanboukouseikagakuka/0000080278.pdf).

### Dataset

The CXR datasets used for developing and evaluating our DL model contain CXRs for the following three categories: normal CXR (NORMAL), non-COVID-19 pneumonia CXR (PNEUMONIA), and COVID-19 pneumonia CXR (COVID). Our DL model was developed using two public (COVIDx and COVID_BIMCV_) and one private (COVID_private_) datasets. One public dataset (COVIDx) was built to accelerate the development of highly accurate and practical deep learning model for detecting COVID-19 cases (https://github.com/lindawangg/COVID-Net/blob/master/docs/COVIDx.md)^15^. The other public dataset (COVID_BIMCV_) was constructed from two public datasets: the PadChest dataset (https://github.com/auriml/Rx-thorax-automatic-captioning)^16^ and BIMCV-COVID19+ dataset (https://github.com/BIMCV-CSUSP/BIMCV-COVID-19)^17^. COVID_private_ was based on the dataset collected from six hospitals previously, and the two public datasets (COVIDx and COVID_BIMCV_) were the same as those in previous studies^18,19^. The details of these datasets are described in the Supplementary material. Compared with the previous study, CXRs were added for COVID_private_ in the current study. The additional CXRs included 37, 7, and 31 cases of NORMAL, PNEUMONIA, and COVID, respectively. COVID_private_ contained 530 CXRs (176 NORMAL, 146 PNEUMONIA, and 208 COVID).

In addition to COVID_private_, CXRs were collected from two other medical institutions. In total, 168 CXRs (80 NORMAL, 37 PNEUMONIA, and 51 COVID) collected from one medical institution (Hospital A) were used for the internal validation of the DL model (as a part of validation set) and for radiologists’ reading practice conducted before the observer study. Moreover, as unseen test set, 180 CXR cases (60 NORMAL, 60 PNEUMONIA, and 60 COVID) collected from another medical institution (Hospital B) were used for the external validation of the DL model and observer study of radiologists.

In the Hospital B, COVID was limited to those diagnosed with COVID-19 pneumonia using RT-PCR, and CXR was obtained after symptom onset. The time of COVID-19 diagnosis was between January 24, 2020, and May 5, 2020. PNEUMONIA was defined as patients clinically diagnosed with bacterial pneumonia that improved with appropriate treatment. Patients who showed no pneumonia on CT or had lung metastasis of malignancy and acute exacerbation of interstitial pneumonia were excluded from PNEUMONIA. NORMAL was defined as the absence of abnormalities in the lung, mediastinum, thoracic cavity, or chest wall on CXR and CT. NORMAL and PNEUMONIA were limited to cases before the summer of 2019 (before the COVID-19 pandemic). The details of the unseen test set collected from the Hospital B are described in the Supplementary material. The inclusion criteria of CXRs in the COVID_private_ and the Hospital A were the same as the previous study^19^.

Table 1 lists the details of each CXR dataset. The 180 cases (as the unseen test set) used for the external validation and reading sessions were adults aged 20 years or older. In the 180 cases, NORMAL included 39 men and 21 women aged 58.1 ± 27.9 years. PNEUMONIA included 43 men and 17 women aged 76.2 ± 20.8 years. The COVID group included 46 men and 14 women aged 53.4 ± 38.6 years.

**Table 1:** Numbers of CXR images in the datasets: COVIDx, COVID_BIMCV_, and COVID_private_, Hospital A, and Hospital B.

Dataset	Total number of CXR images	Number of CXR images of NORMAL	Number of CXR images of PNEUMONIA	Number of CXR images of COVID
COVIDx	14,258	8066	5575	617
COVID_BIMCV_	11,253	8799	979	1475
COVID_private_	530	176	146	208
Hospital A	168	80	37	51
Hospital B	180	60	60	60

All cases of PNEUMONIA were bacterial pneumonia in COVID_private_, Hospital A, and Hospital B. Abbreviations: CXR, chest X-ray; COVIDx, public dataset used for COVID-Net; COVID_BIMCV_, public dataset obtained from the PadChest and BIMCV-COVID19+ datasets; COVID_private_, private dataset collected from six hospitals. Hospital A, dataset collected for internal validation; Hospital B, dataset collected for external validation. Hospitals A and B were not included in the six hospitals where COVID_private_ data were collected.

### Deep learning model

**Figure 1:**
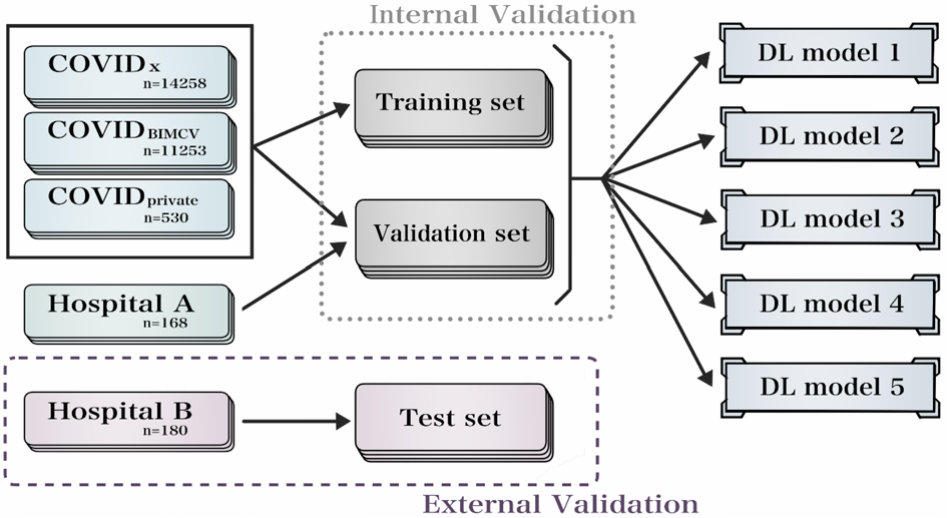
Schematic illustration of dataset splitting and model training for our DL model. Abbreviation: DL, deep learning; COVIDx, public dataset used for COVID-Net; COVID_BIMCV_, public dataset obtained from the PadChest and BIMCV-COVID19+ datasets; COVID_private_, private dataset collected from six hospitals; Hospital A, dataset collected for internal validation and radiologist’s practice before the observer study; Hospital B, dataset collected for external validation.

Our EfficientNet-based DL model was constructed in the same manner as described in previous papers^18,19^. Figure 1 shows a schematic of the construction of the DL model. There are two major differences in the DL model construction between the present study and previous studies; one is that the 168 CXRs collected from Hospital A were used for internal validation as a part of the validation set, and the other is that the 180 CXRs collected from Hospital B were used for external validation as the unseen test set. The DL model development set included two public datasets, COVID_private_, and 168 CXRs collected from Hospital A. Five different random divisions of the training and validation sets were created from the development set. In the division, 300, 300, and 90 images were randomly selected as the validation set from COVIDx, COVID_BIMCV_, and COVID_private_, respectively. The remaining images of COVIDx, COVID_BIMCV_, and COVID_private_ were used as the train set. In addition, all the 168 CXRs collected from Hospital A were used for the validation set. Model training and internal validation of diagnostic performance were performed for the training set and validation set, respectively. The training of our DL model is also described in the Supplementary material.

The inference results of the DL model were calculated using an ensemble of five trained models. For the 180 CXRs of the external validation, an average of the probabilities obtained from the five trained models was calculated as the inference results of the DL model to evaluate the diagnostic performance of the DL model and to provide supporting information for radiologists during the observer study.

The DL model calculated the probability of NORMAL, PNEUMONIA, or COVID for each CXR, with a total of 100%. We also created images using Grad-CAM and Grad-CAM++ as explainable artificial intelligence, which visualized the reasoning for the diagnosis of the DL model^20,21^. Grad-CAM and Grad-CAM++ images were used for the observer study. Min–max normalization with a linear transformation was performed on the original Grad-CAM and Grad-CAM++ images.

### Observer study

Eight radiologists (with 5–20 years of experience in diagnostic radiology) performed the observer study at two medical facilities. For the 180 CXRs collected from Hospital B, each radiologist performed two reading sessions over a period of more than 1 month. One reading session was performed with reference to CXRs only, and the other was performed with reference to both CXRs and the results of the DL model. The order of the two sessions was randomly selected to reduce bias. The eight radiologists scored the probabilities of NORMAL, PNEUMONIA, and COVID on a 100% scale. In the reading session with the DL model, the radiologists referred to the probabilities of NORMAL, PNEUMONIA, and COVID calculated using the DL model. If there was any uncertainty regarding the probabilities of the DL model, the results of Grad-CAM and Grad-CAM++ were available. Images of the 168 CXRs collected from Hospital A were also processed with Grad-CAM and Grad-CAM++ , and the diagnosis of the DL model and images of Grad-CAM and Grad-CAM++ of the 168 CXRs were presented to the radiologists for practice sessions before each reading session. Eight radiologists were taught how to interpret the Grad-CAM and Grad-CAM++ images before the observer study. There was no time limit for reading and practice sessions. Prior to the reading sessions, only the approximate frequencies of the three categories were presented to the radiologists and no other clinical information was provided. Our novelties in this study were to investigate whether radiologists changed their diagnosis by referring to our DL model of CXR and whether the diagnostic performance of radiologists was significantly improved.

### *Evaluation of Grad-CAM*++ *images*

After the observer study, one senior radiologist visually evaluated the 180 Grad-CAM++ images in the test set. The visual evaluation of the Grad-CAM++ images was performed on the images that were accurately diagnosed by the DL. The radiologist visually examined the CXR and Grad-CAM++ images and determined whether the Grad-CAM++ images were typical or understandable. The typical Grad-CAM++ images were described in Supplementary material. If abnormal findings on CXR images were highlighted on Grad-CAM++ images, the cases were considered understandable by the radiologist. In addition, for COVID, the radiologist counted the number of Grad-CAM++ images with highlighted regions outside the lung area.

### Statistical analyses

We evaluated the diagnostic performance of the DL model alone and compared the results between reading sessions with and without the DL model. The evaluation metrics were accuracy, sensitivity, specificity, and area under the curve (AUC) in the receiver operating characteristics. Because three-category classification was performed, these metrics were calculated class-wise (one-vs-rest), except for accuracy. For the AUC, multi-reader multi-case statistical analysis was used to statistically analyze the results of the eight radiologists. MRMCaov was used for the statistical analyses^22^. Although MRMCaov is a statistical method designed for binary classification of two categories, this study was designed to diagnose three categories: NORMAL, PNEUMONIA, and COVID. Therefore, the three-category classification was divided into three binary classifications (one-vs-rest): (1) NORMAL versus PNEUMONIA or COVID, (2) PNEUMONIA versus NORMAL or COVID, and (3) COVID versus NORMAL or PNEUMONIA. We then compared the class-wise AUC of the eight radiologists between reading sessions with and without the DL model. The difference in the AUC was statistically tested using MRMCaov. Because it was necessary to integrate the results from the eight radiologists, the class-wise MRMCaov was used in the present study. To control the family-wise error rate, Bonferroni correction was used; a *p* value less than 0.01666 was considered statistically significant. R (version 4.1.2) was used for the statistical analysis.

## Results

**Figure 2:**
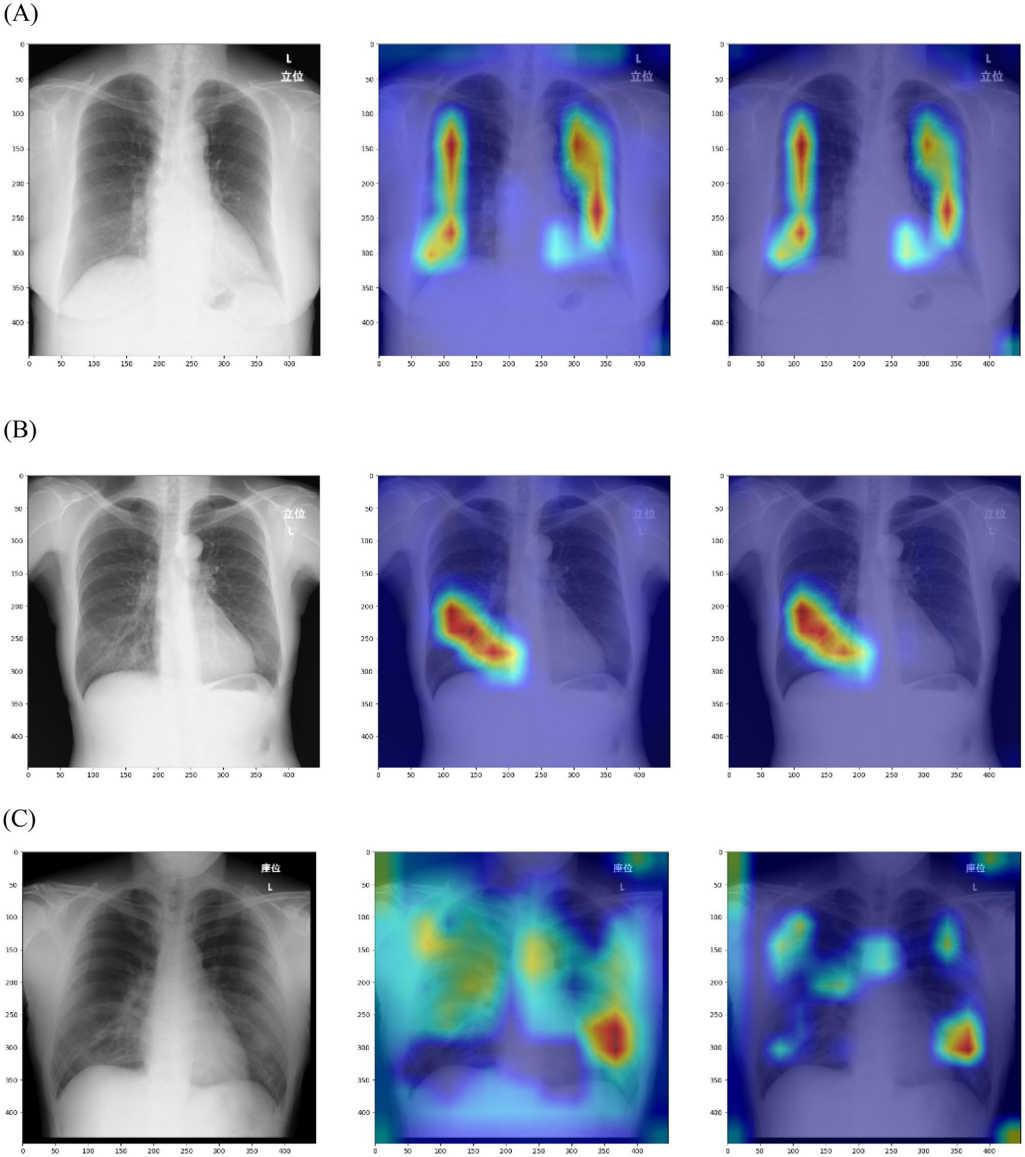
Results of Grad-CAM and Grad-CAM++ for our DL model. (**A**) NORMAL, (**B**) PNEUMONIA, and (**C**) COVID. Each row consists of CXR images collected from Hospital A and the Grad-CAM and Grad-CAM++ results. One trained DL model was used for Grad-CAM and Grad-CAM++ . Left column, original CXR image; middle column, result of Grad-CAM; right column, results of Grad-CAM++ . Abbreviations: DL, deep learning; CXR, chest X-ray.

Figure 2 shows examples of CXR, Grad-CAM, and Grad-CAM++ images from NORMAL, PNEUMONIA, and COVID. As shown in Fig. 2, in the images of Grad-CAM and Grad-CAM++ from NORMAL, there was often a relatively symmetrical region of interest in the lung fields. In PNEUMONIA, the region of interest was observed in the unilateral lung field in most cases, which was consistent with an abnormal shadow caused by pneumonia. COVID tended to show regions of interest in both the lungs and mediastinum.

**Figure 3:**
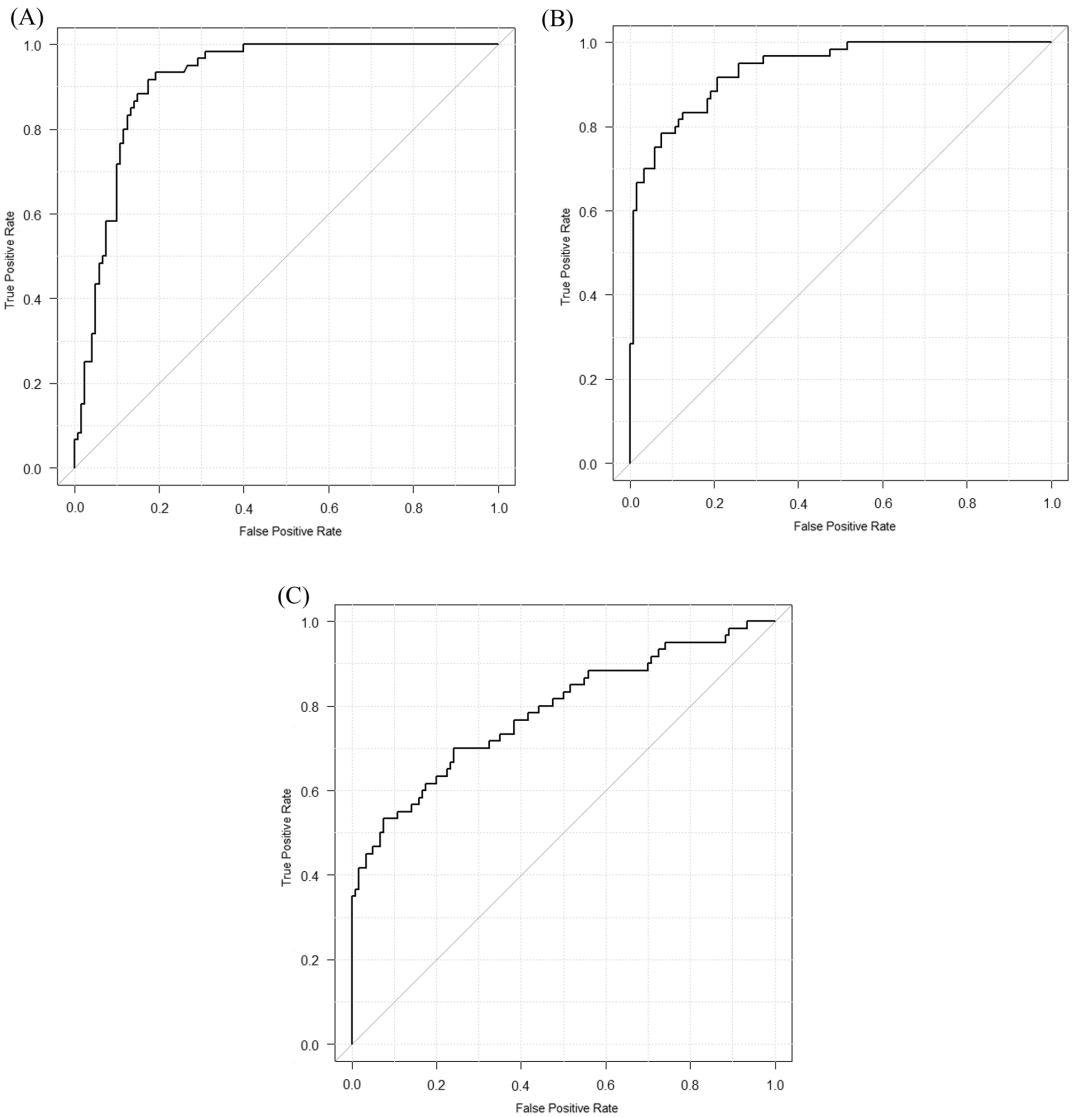
Class-wise receiver operating characteristics curves of our DL model in external validation. (**A**) NORMAL versus PNEUMONIA or COVID, (**B**) PNEUMONIA versus NORMAL or COVID, and (**C**) COVID versus NORMAL or PNEUMONIA. Abbreviation: DL, deep learning.

**Figure 4:**
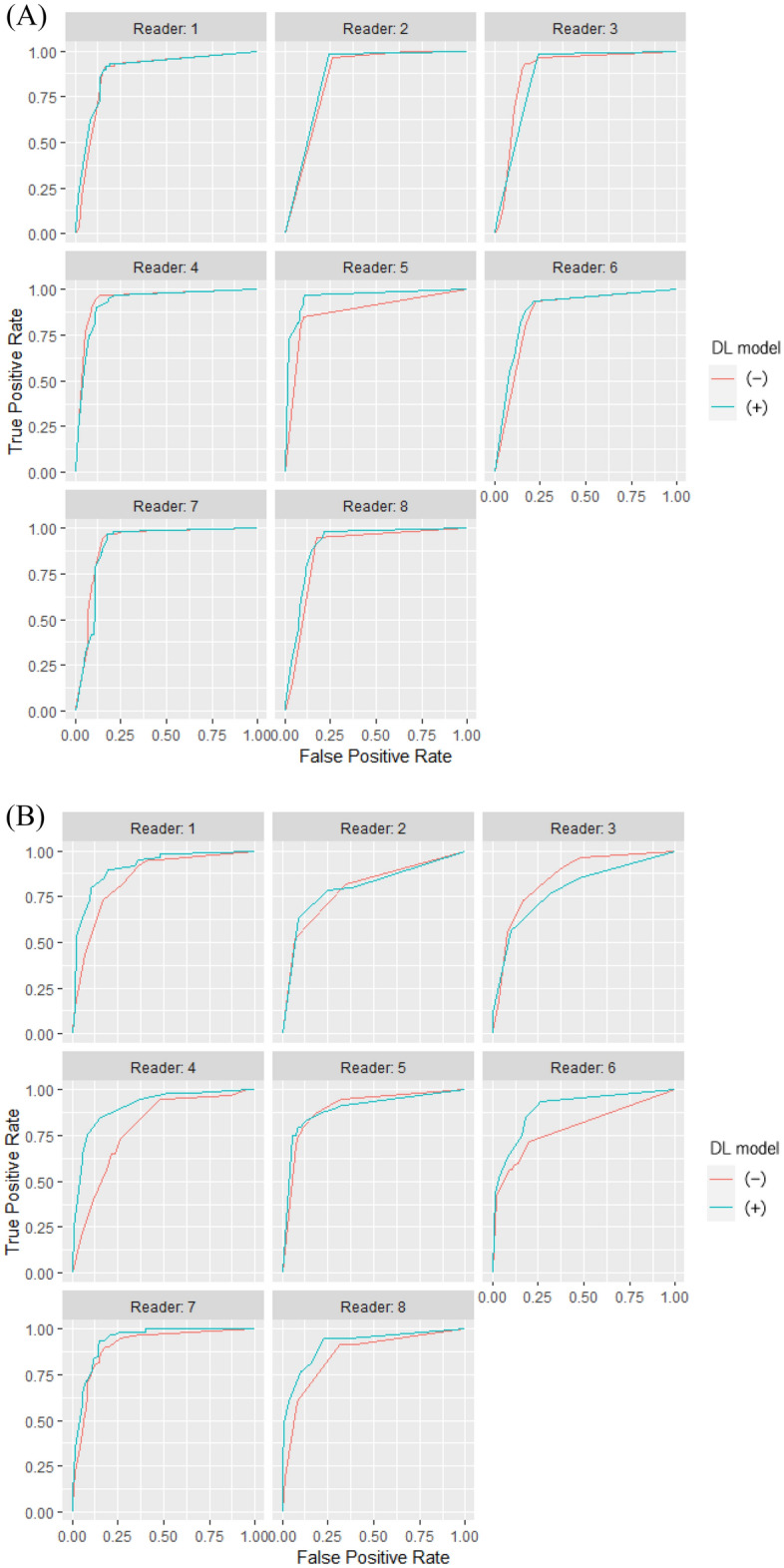

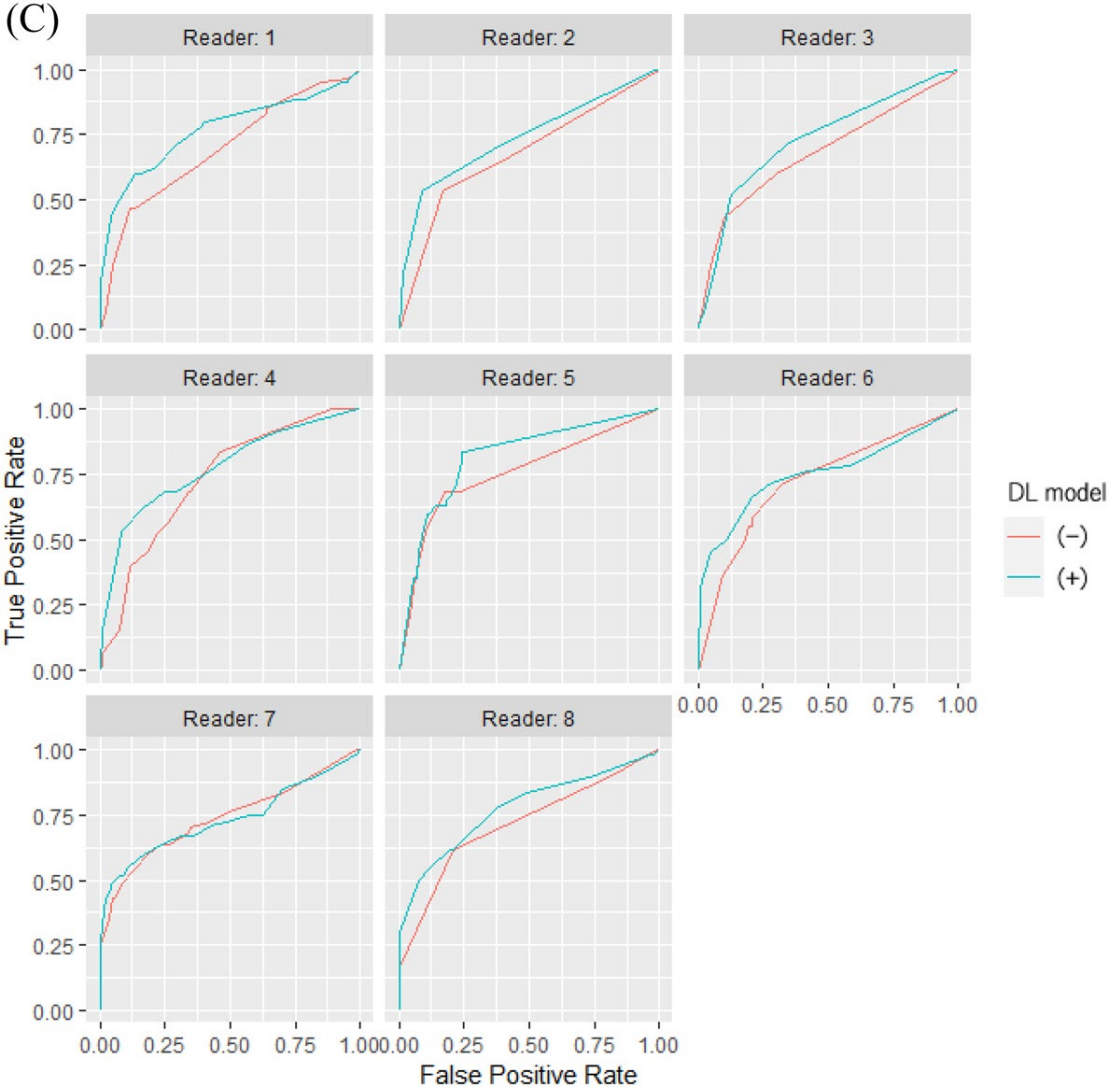
Class-wise receiver operating characteristics curves of eight radiologists with and without our DL model in observer study. (**A**) NORMAL versus PNEUMONIA or COVID, (**B**) PNEUMONIA versus NORMAL or COVID, and (**C**) COVID versus NORMAL or PNEUMONIA. The blue and red lines represent the receiver operating characteristic curves of the radiologists with and without our DL model, respectively. Abbreviation: DL, deep learning.

Table 2 shows the sensitivity, specificity, accuracy, and AUC of the DL model and eight radiologists with and without the DL model. Here, the three types of binary classifications (one-vs-rest) were defined as follows: A, “NORMAL versus PNEUMONIA or COVID”; B, “PNEUMONIA versus NORMAL or COVID”; and C, “COVID versus NORMAL or PNEUMONIA.” Fig. 3 shows the receiver operating characteristics curves of our DL model alone for the three types of binary classifications. Figure 4 shows the receiver operating characteristics curves of eight radiologists with and without the DL model.

**Table 2:** Class-wise sensitivity, specificity, AUC, and 3-category classification accuracy of our DL model alone and eight radiologists with and without our DL model.

		Sensitivity			Specificity			accuracy	AUC		
		A	B	C	A	B	C		A	B	C
Reader1	DL (−)	0.883	0.733	0.467	0.850	0.825	0.867	0.694	0.882	0.852	0.696
	DL (+)	0.833	0.783	0.600	0.867	0.900	0.842	0.739	0.895	0.912	0.768
Reader2	DL (−)	0.967	0.517	0.533	0.742	0.933	0.833	0.672	0.860	0.797	0.679
	DL (+)	0.983	0.633	0.533	0.758	0.908	0.908	0.717	0.871	0.802	0.738
Reader3	DL (−)	0.900	0.733	0.467	0.850	0.825	0.875	0.700	0.891	0.855	0.682
	DL (+)	0.983	0.567	0.517	0.758	0.900	0.875	0.689	0.876	0.792	0.730
Reader4	DL (−)	0.817	0.650	0.517	0.933	0.775	0.783	0.661	0.941	0.792	0.731
	DL (+)	0.833	0.750	0.633	0.892	0.908	0.808	0.739	0.928	0.911	0.777
Reader5	DL (−)	0.817	0.783	0.667	0.908	0.892	0.833	0.756	0.877	0.900	0.757
	DL (+)	0.833	0.800	0.633	0.917	0.900	0.817	0.756	0.954	0.895	0.816
Reader6	DL (−)	0.867	0.583	0.550	0.808	0.883	0.808	0.667	0.867	0.792	0.725
	DL (+)	0.817	0.633	0.667	0.858	0.908	0.792	0.706	0.886	0.896	0.755
Reader7	DL (−)	0.783	0.767	0.600	0.892	0.883	0.800	0.717	0.915	0.905	0.736
	DL (+)	0.783	0.767	0.600	0.892	0.883	0.800	0.717	0.903	0.938	0.733
Reader8	DL (−)	0.883	0.600	0.617	0.842	0.917	0.792	0.700	0.882	0.856	0.718
	DL (+)	0.783	0.767	0.617	0.883	0.892	0.808	0.722	0.912	0.919	0.776
Mean ± SD	DL (−)	0.865 ± 0.058	0.671 ± 0.097	0.552 ± 0.072	0.853 ± 0.060	0.867 ± 0.053	0.824 ± 0.034	0.696 ± 0.031	0.889 ± 0.027	0.844 ± 0.046	0.716 ± 0.028
Mean ± SD	DL (+)	0.856 ± 0.081	0.713 ± 0.088	0.600 ± 0.051	0.853 ± 0.061	0.900 ± 0.009	0.723 ± 0.041	0.723 ± 0.021	0.903 ± 0.028	0.883 ± 0.055	0.762 ± 0.029
DL model		0.750	0.783	0.667	0.900	0.917	0.783	0.733	0.913	0.937	0.786

*AUC* area under the curve, *DL* deep learning, *SD* standard deviation; A, NORMAL versus PNEUMONIA or COVID; B, PNEUMONIA versus NORMAL or COVID; C, COVID versus NORMAL or PNEUMONIA.

The three-category classification accuracy of the DL model was 0.733 (132/180). The 95% confidence intervals of class-wise AUC of the DL model were as follows: A, 0.872–0.955; B, 0.903–0.972; and C, 0.711–0.862. The mean accuracy of radiologists without the DL model was 0.696 ± 0.031 (range, 0.667 [120/180]–0.756 [136/180]). Their class-wise AUCs without the DL model were as follows: A, 0.889 ± 0.027 (0.860–0.941); B, 0.844 ± 0.046 (0.792–0.905); and C, 0.716 ± 0.028 (0.679–0.757). The mean accuracy of radiologists with the DL model was 0.723 ± 0.021 (range, 0.689 [124/180]–0.756 [136/180]). Their class-wise AUCs with the DL model were as follows: A, 0.903 ± 0.028 (0.871–0.954); B, 0.883 ± 0.055 (0.792–0.938); and C, 0.762 ± 0.029 (0.730–0.816). The accuracy of our DL model was better than that of six radiologists without the DL model.

Table 3 shows the averaged AUC of senior and junior radiologists with and without our DL model. The numbers of senior and junior radiologists were five and three, respectively. According to the Table 3, in both senior and junior radiologists, the difference of averaged class-wise AUC for C (“COVID versus NORMAL or PNEUMONIA”) between with and without the DL model was larger than those for A and B.

**Table 3:** Averaged AUC of senior and junior radiologists with and without our DL model.

		AUC		
		A	B	C
Senior radiologists Mean ± SD	DL (−)	0.901 ± 0.027	0.861 ± 0.046	0.720 ± 0.031
Junior radiologists Mean ± SD	DL (−)	0.870 ± 0.011	0.815 ± 0.036	0.707 ± 0.025
Senior radiologists Mean ± SD	DL (+)	0.911 ± 0.030	0.890 ± 0.057	0.765 ± 0.035
Junior radiologists Mean ± SD	DL (+)	0.890 ± 0.021	0.872 ± 0.062	0.756 ± 0.019

The numbers of senior and junior radiologists are five and three, respectively. *AUC* area under the curve, *DL* deep learning, *SD* standard deviation; A, NORMAL versus PNEUMONIA or COVID; B, PNEUMONIA versus NORMAL or COVID; C, COVID versus NORMAL or PNEUMONIA.

**Figure 5:**
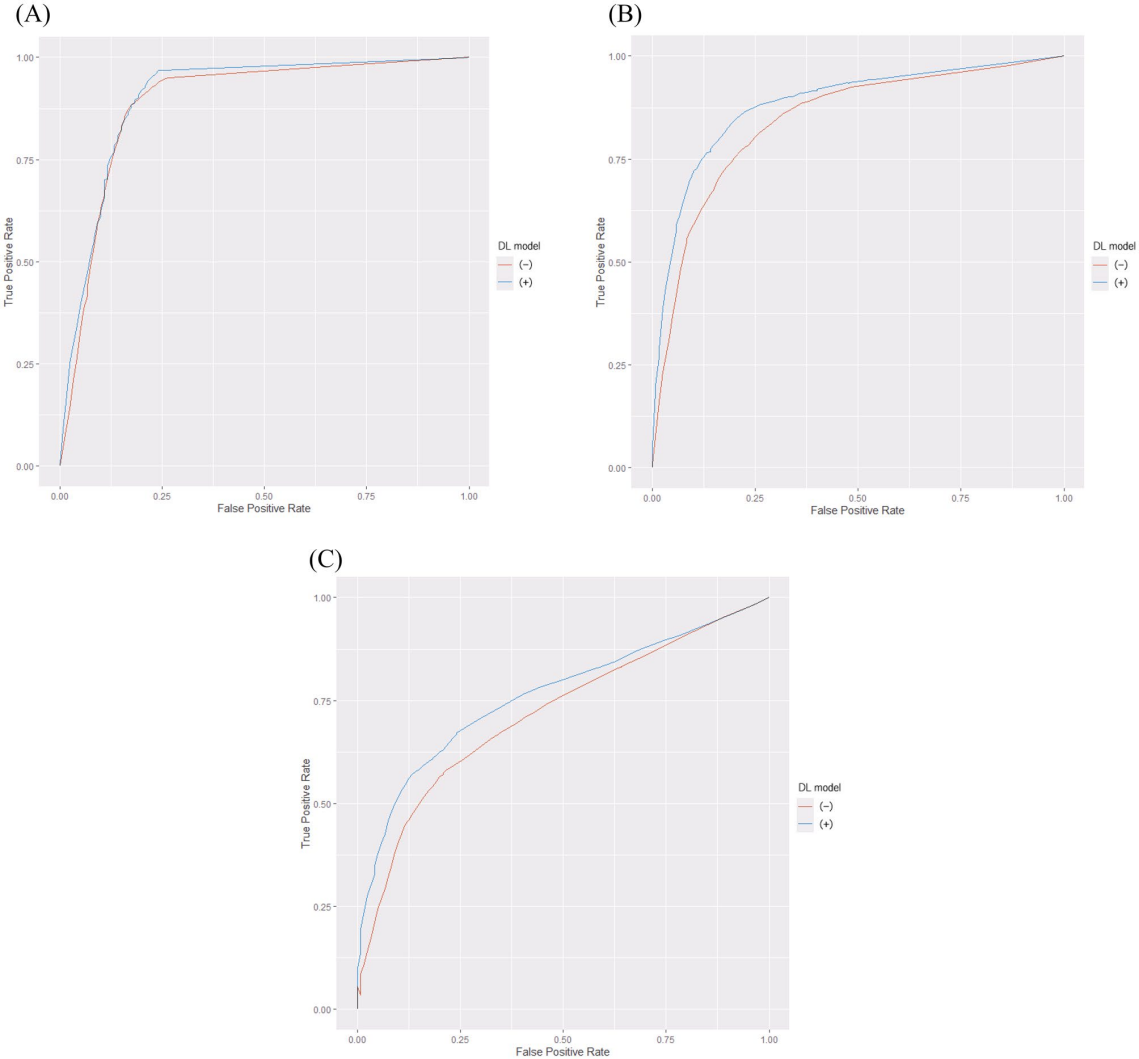
Class-wise receiver operating characteristics curves obtained by integration of reading session results of eight radiologists with and without our DL model. Note: (**A**) NORMAL versus PNEUMONIA or COVID, (**B**) PNEUMONIA versus NORMAL or COVID, (**C**) COVID versus NORMAL or PNEUMONIA. The blue and red lines represent the integrated receiver operating characteristics curves of radiologists with and without our DL model, respectively. Abbreviation: DL, deep learning.

We integrated the results of eight radiologists with and without the DL model using the software MRMCaov and compared the class-wise AUC of radiologists between reading sessions with and without the DL model. The results of MRMCaov showed that in the classification C (COVID versus NORMAL or PNEUMONIA), there were significant differences in AUC between the radiologists with and without the DL model (*p* = 0.0038). In classifications A and B, there were no significant differences in the AUC between the radiologists with and without the DL model (*p* = 0.2396 and 0.1190, respectively). Figure 5 shows the class-wise receiver operating characteristics curves of the integrated results of eight radiologists with and without the DL model.

Table 4 shows the results of visual evaluation of the Grad-CAM++ images. The ratio of the typical or understandable Grad-CAM++ images was 0.932 (123/132). The ratio of Grad-CAM++ images highlighted outside the lung area was 0.200 (8/40) for COVID.

**Table 4:** Results of the visual evaluation of Grad-CAM +  + images in the unseen test set.

	Number	Ratio
Accurate diagnosis by DL model	132	0.733 (132/180)
Typical or understandable Grad-CAM++ images	123	0.932 (123/132)
Grad-CAM++ images highlighted outside the lung area for COVID	8	0.200 (8/40)

The value in the parenthesis means numerator and denominator for the ratio. *DL* deep learning.

## Discussion

In this study, eight radiologists performed the reading sessions with and without the DL model, and the results were compared and analyzed using multi-reader multi-case statistical analysis. The diagnostic performance of the DL model alone was also evaluated. Our DL model achieved a higher accuracy and AUC than the majority of the eight radiologists without the DL model. Furthermore, the results of the statistical analysis showed that radiologists’ diagnostic performance was significantly improved by the DL model in diagnosing COVID-19 on CXR.

Based on the results of the receiver operating characteristics analysis with MRMCaov, there was a significant difference in AUC of radiologists between with and without the DL model for “C: COVID versus NORMAL or PNEUMONIA” (*p* = 0.0038). However, there was no significant difference for “A: NORMAL versus PNEUMONIA or COVID” and “B: PNEUMONIA versus NORMAL or COVID.” One possible reason for these results may be that radiologists have less experience in reading COVID than NORMAL or PNEUMONIA. Based on these results, the DL model may be more useful for medical doctors in other fields with less experience in reading COVID.

Because the DL model alone had a higher diagnostic performance than the majority of the eight radiologists, it may be possible to apply the DL model to COVID-19 diagnosis on CXR for screening and other purposes. This DL model of CXRs may be useful, especially in areas where medical resources are limited.

In a previous study, our DL model was significantly superior to radiologists in diagnosing COVID-19 pneumonia^19^. However, the DL model was not evaluated as computer-aided diagnosis system in the previous study. On the other hand, because the reading sessions of the present study were conducted by radiologists with and without the DL model, this is more similar to the situation of practical clinical use of the DL model. In addition, the previous study had the disadvantage that it was performed by internal validation. The current study was performed using external validation, which generally produces more reliable results than internal validation. Rangarajan et al.^23^ also performed external validation of the DL model for COVID-19 diagnosis. They pointed out that their DL model may complement COVID diagnosis on CXR. Although the study by Rangarajan et al. is similar to our study, the classification targets and method of statistical analysis are different from ours.

To the best of our knowledge, there are no studies in which three-category classification (including COVID) was performed using DL models and external validation. This study is the first to evaluate the generalizability of the DL model in a three-category classification. Several studies have compared the diagnostic performance of the DL model with that of radiologists for COVID-19 on CXR^12–14^. They reported that the AUC and accuracy of the DL model tended to exceed those of radiologists in most cases. For example, Wehbe et al.^14^ compared the diagnostic performance between their DL model and two radiologists in the diagnosis of COVID-19 positive and COVID-19 negative. Their DL had a significantly higher sensitivity (71%) than that of one radiologist (60%) and a significantly higher specificity (92%) than that of two radiologists (75% and 84%, respectively).

RT-PCR is the most commonly used test to detect COVID-19, but its sensitivity is not significantly high. One study reported that the sensitivity of RT-PCR is approximately 71%^3^. RT-PCR is also time consuming and often difficult to perform in small medical facilities. This is particularly true in developing countries. In contrast, CXR is a simple imaging examination. The disadvantage of CXR is that its diagnostic performance depends on the reader’s ability. The sensitivity and specificity of our DL model were relatively high for the three types of target classification. Therefore, it may be possible to increase the usefulness of CXR as an alternative or complementary test to RT-PCR.

One of the reasons why we evaluated our DL model by external validation is that it is difficult to evaluate the DL model accurately using public datasets. Garcia Santa Cruz et al. pointed out that public datasets contain undetected bias^24^. When these datasets are used for internal validation, there is a risk of overestimation of the diagnostic performance of the DL model. Therefore, we attempted to mitigate these biases using external validation.

Our study has some limitations. First, the CXRs in this study were obtained from large-sized hospitals, and good-quality CXRs were used. Therefore, we did not evaluate the usefulness of our DL model on poor-quality CXRs. Second, we conducted an observer study for CXRs with normal, non-COVID-19 pneumonia, and COVID-19 pneumonia. Because we excluded CXRs with other lung diseases, we could not assess the usefulness of our DL model for these images.

In conclusion, our DL model alone showed better diagnostic performance than most of the eight radiologists in the external validation of the three-category classifications of normal, non-COVID-19 pneumonia, and COVID-19 pneumonia. In addition, our DL model significantly improved the diagnostic performance of the eight radiologists in COVID-19 pneumonia versus normal or non-COVID-19 pneumonia.

### Supplementary Information

Supplementary Information.

## Data Availability

The source code of our DL model and its related data are available from the following URL of GitHub: https://github.com/jurader/covid19_xp_efficientnet.
