# Supplementary material for: Computer-aided diagnosis of chest X-ray for COVID-19 diagnosis in external validation study by radiologists with and without deep learning system
Source: Sci Rep. 2023 Oct 16;13:17533. doi: 10.1038/s41598-023-44818-9 (PMC10579343; doi:10.1038/s41598-023-44818-9)
Supplement: Supplementary file 1 — Supplementary Information. [file 41598_2023_44818_MOESM1_ESM.docx]

**Supplementary Material**

**The details of the private dataset and the two public datasets used in the previous studies**

Our deep learning (DL) model was developed using two public and one private datasets (1-4). One public dataset was the COVIDx dataset (1). The other public dataset was constructed from two public datasets: the PadChest dataset (2) and BIMCV-COVID19+ dataset (3). The second public dataset was referred to as COVID_BIMCV_. These two public datasets were the same as those in the previous studies (4). The private dataset was based on the dataset collected from six hospitals previously (4). Hereafter, we will refer to the previous private dataset COVID_prev_private_.

The total number of CXR images was 14258, 11253, and 455 in the COVIDx, COVID_BIMCV_, and COVID_prev_private_ datasets, respectively. The number of CXR images of the NORMAL, PNEUMONIA, and COVID was 139, 139, and 177 in the COVID_prev_private_ dataset, respectively.

For the current study, new CXR images was added to COVID_prev_private_. After the addition, the number of CXR images was 530 (This dataset is referred to as COVID_private_ in the main text). The following Figure shows the relationship of the datasets between the previous study (4) and the current study.

Relationship between previous study and current study for the private dataset of CXR.


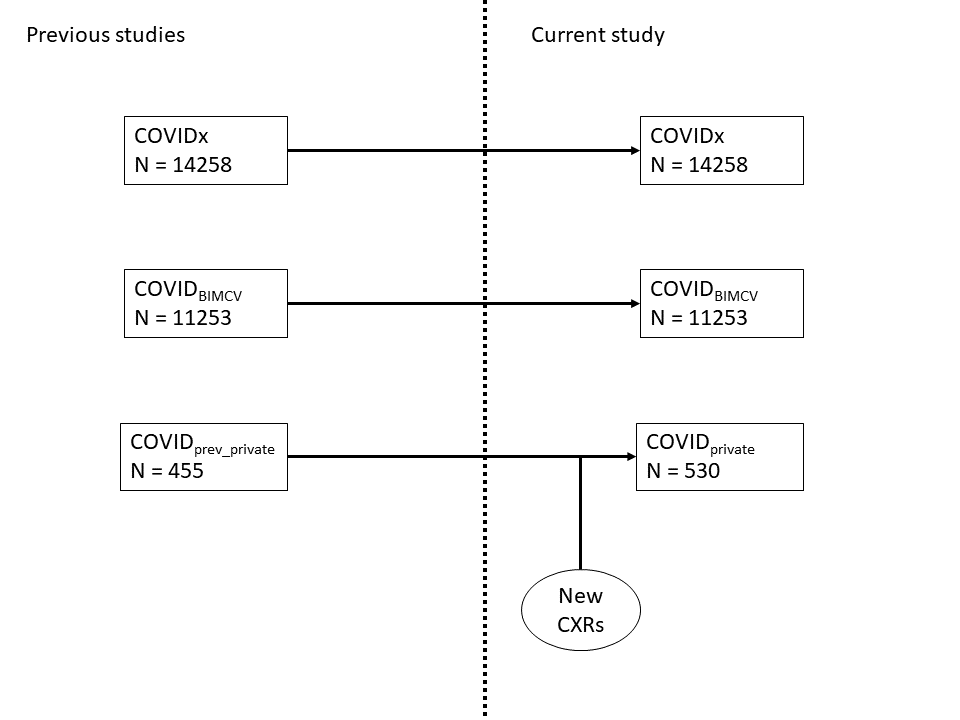


**The details of dataset in the Hospital B for external validation (unseen test set)**

The following inclusion criteria were used for COVID in the Hospital B. The COVID-19 pneumonia who met the inclusion criteria was sequentially included in this study. Although CT examinations were performed for the COVID-19 pneumonia in the Hospital B, the CT examinations were not used in the inclusion criteria.

- Age >= 20
- COVID-19 pneumonia was diagnosed with RT-PCR (RT-PCR positive)
- CXR was obtained after symptom onset

PNEUMONIA in the Hospital B was defined as patients clinically diagnosed with bacterial pneumonia that improved with appropriate treatment. Mainly, result of sputum culture and/or clinical course (effectiveness of antibiotic response) were used for the confirmation of PNEUMONIA in the Hospital B. Fungal pneumonia and viral pneumonia were carefully excluded from PNEUMONIA.

**Training of our DL model**

As in the previous study (4), our DL model was based on EfficientNet (5). The layers of EfficientNet were sorted in the order of image processing, and trainable parameters was frozen for several layers of EfficientNet. Next, the global averaging pooling layer, fully-connected layer, and dropout layer were added, after the convolution layers of EfficientNet. For the 3-category classification, the final fully-connected layer was added after the dropout layer. Activation functions of the first and second fully-connected layer were rectified linear unit and softmax, respectively.

The training of our DL model was performed using a workstation with a discrete GPU (Nvidia RTX 3090, RAM 24 GB). Python (version 3.8, http://www.python.org/) was used as the programing language, and Keras (version 2.4.3, http://keras.io/) and TensorFlow (version 2.4.0, http://tensorflow.org/) were used as deep learning frameworks.

**Practice sessions for Grad-CAM and Grad-CAM++**

The diagnosis of the DL model and the Grad-CAM and Grad-CAM++ images of the 168 CXRs collected from Hospital A were presented to the radiologists for practice sessions.

In addition to the CXRs and their Grad-CAM and Grad-CAM++ results, the following instruction was given to the radiologists during the practice sessions.

***** Instruction *****

At the end of this instruction, there is explanation for typical cases of NORMAL, PNEUMONIA, and COVID.

Each case of practice sessions has CXR, Grad-CAM and Grad-CAM++ images. The left image is the original CXR image, the middle image is highlighted with Grad-CAM, and the right image is highlighted with Grad-CAM++. The Grad-CAM and Grad-CAM++ images were created using different algorithms.

If you are interested in Grad-CAM, and Grad-CAM++, please refer to the following citations.

- Grad-CAM+：Selvaraju, R. R. et al. Grad-CAM: Visual Explanations from Deep Networks via Gradient-Based Localization. Int. J. Comput. Vis. 128, 336–359 (2020) (https://openaccess.thecvf.com/content_ICCV_2017/papers/Selvaraju_Grad-CAM_Visual_Explanations_ICCV_2017_paper.pdf)
- Grad-CAM++：A. Chattopadhyay et al., " Grad-CAM++: Improved Visual Explanations for Deep Convolutional Networks," arXiv preprint arXiv:1710.11063v3, 2018（https://arxiv.org/pdf/1710.11063.pdf）

NORMAL case:

In NORMAL case, the Gram-CAM and Grad-CAM++ images are relatively symmetrical, with highlighted regions extending vertically across the lung fields.

PNEUMONIA case:

In PNEUMONIA case, color concentration is focused on the area affected by pneumonia. Typically, there is a lack of symmetry.

COVID case:

In COVID case, highlighted regions frequently extend across both lungs. Furthermore, compared to NORMAL and PNEUMONIA cases, there is a tendency for colorization along the contours of the lung fields and outside the lung area.

**References**

1. Wang L, Lin ZQ, Wong A. COVID-Net: a tailored deep convolutional neural network design for detection of COVID-19 cases from chest X-ray images. Sci Rep. 2020 Nov 11;10(1):19549. doi: 10.1038/s41598-020-76550-z.

2. Bustos A, Pertusa A, Salinas JM, de la Iglesia-Vayá M. PadChest: A large chest x-ray image dataset with multi-label annotated reports. Med Image Anal. 2020 Dec;66:101797. doi: 10.1016/j.media.2020.101797.

3. Vayá, MDLI, Saborit JM, Montell JA, et al. BIMCV COVID-19+: a large annotated dataset of RX and CT images from COVID-19 patients. arXiv. 2020. http://arxiv.org/abs/2006.01174.

4. Nishio M, Kobayashi D, Nishioka E, et al. Deep learning model for the automatic classification of COVID-19 pneumonia, non-COVID-19 pneumonia, and the healthy: a multi-center retrospective study. Sci Rep. 2022 May 17;12(1):8214. doi: 10.1038/s41598-022-11990-3.

5. Tan M, Le QV. EfficientNet: Rethinking Model Scaling for Convolutional Neural Networks. *36th Int. Conf. Mach. Learn. ICML 2019* 2019-June. 2019:10691–10700.
